# Internal and External Load Profile during Beach Invasion Sports Match-Play by Electronic Performance and Tracking Systems: A Systematic Review

**DOI:** 10.3390/s24123738

**Published:** 2024-06-08

**Authors:** Pau Vaccaro-Benet, Carlos D. Gómez-Carmona, Joaquín Martín Marzano-Felisatti, José Pino-Ortega

**Affiliations:** 1 Department of Physical Activity and Sport, Faculty of Sport Sciences, University of Murcia, 30720 Murcia, Spain; p.vaccarobenet@um.es (P.V.-B.); josepinoortega@um.es (J.P.-O.); 2 BioVetMed & SportSci Research Group, University of Murcia, 30100 Murcia, Spain; 3 Training Optimization and Sports Performance Research Group (GOERD), Department of Didactics of Music Plastic and Body Expression, Faculty of Sport Science, University of Extremadura, 10003 Caceres, Spain; 4 Research Group in Sports Biomechanics (GIBD), Department of Physical Education and Sports, Faculty of Physical Activity and Sport Sciences, Universitat de València, 46010 Valencia, Spain; joaquin.marzano@uv.es

**Keywords:** athlete monitoring, global positioning systems, time–motion analysis, high-intensity interval training, speed, team sports

## Abstract

Beach variants of popular sports like soccer and handball have grown in participation over the last decade. However, the characterization of the workload demands in beach sports remains limited compared to their indoor equivalents. This systematic review aimed to: (1) characterize internal and external loads during beach invasion sports match-play; (2) identify technologies and metrics used for monitoring; (3) compare the demands of indoor sports; and (4) explore differences by competition level, age, sex, and beach sport. Fifteen studies ultimately met the inclusion criteria. The locomotive volumes averaged 929 ± 269 m (average) and 16.5 ± 3.3 km/h (peak) alongside 368 ± 103 accelerations and 8 ± 4 jumps per session. The impacts approached 700 per session. The heart rates reached 166–192 beats per minute (maximal) eliciting 60–95% intensity. The player load was 12.5 ± 2.9 to 125 ± 30 units. Males showed 10–15% higher external but equivalent internal loads versus females. Earlier studies relied solely on a time–motion analysis, while recent works integrate electronic performance and tracking systems, enabling a more holistic quantification. However, substantial metric intensity zone variability persists. Beach sports entail intermittent high-intensity activity with a lower-intensity recovery. Unstable surface likely explains the heightened internal strain despite moderately lower running volumes than indoor sports. The continued integration of technology together with the standardization of workload intensity zones is needed to inform a beach-specific training prescription.

## 1. Introduction

Beach sports have also experienced an increase in participation during the last decade [1]. Following the sports modalities classification realized by Read and Edwards [2], beach sports could be classified as: (a) invasion sports (e.g., soccer and handball), (b) net and wall sports (e.g., volleyball and pickleball), and (c) striking/fielding games (e.g., softball and baseball). Specifically, invasion sports consist of invading the opponent’s territory and scoring a goal or point depending on time [3]. Whilst conventional invasion team sports like soccer, rugby, or handball have been highly monitored and described in the sport sciences area [4,5,6], beach sports variants presented a lack of research. In this sense, precisely quantifying the external and internal loads that athletes are exposed to during the training and competition context has demonstrated their fundamental utility for injury prevention and performance optimization in sports settings [7]. This highlights the need for workload monitoring also in beach sports disciplines, which entail unique demands compared to their indoor equivalents [8].

The workload imposed on athletes can be broadly categorized into two components: external load and internal load [9]. External load represents the mechanical and locomotor actions completed during training or competition, which is quantified through variables like total distance, accelerations/decelerations, jumps, and impacts [10,11]. On the other hand, internal load refers to the relative physiological and psychological stress elicited by the activity, commonly measured via metrics including heart rate, blood lactate concentration, and subjective ratings of perceived exertion scales in different formats (CR-10, CR-100, or 6–20) [12,13].

In beach sports, the time–motion analysis, microtechnologies, or heart rate monitors have been utilized for workload monitoring purposes [14,15]. The most commonly used technology for external workload quantification is global navigation satellite systems (GNSSs), particularly global positioning systems (GPSs) of the American government [16]. Subsequently, local positioning systems (LPSs) were developed to improve the GNSS signal where the satellite coverage is deficient, changing the satellites by antennas around the court [17]. Moreover, microtechnologies (accelerometers, gyroscopes, and magnetometers) have been incorporated to improve the workload monitoring in high-intensity actions with no locomotion (e.g., jumps and collisions) [11].

In this sense, new devices called electronic performance and tracking systems (EPTSs) have been developed that include tracking technologies, microtechnologies, and wireless technologies (e.g., Ant+, Bluetooth, and Wi-Fi) to connect external sensors (e.g., heart rate, muscle oximeters, and blood lactate) and provide a holistic view of internal and external workload demands on athletes [18]. These devices allow the gathering of external load metrics, including the total distance, distance in different speed zones, accelerations/decelerations in different intensity zones, peak speeds, and number of jumps or impacts exceeding given g-forces, amongst others [5]. When coupled with the simultaneous heart rate or rate of perceived exertion values, they permit quantifying both external and internal loads of beach sports matches and training drills in a holistic and non-invasive way [13].

Beach sports present specific characteristics compared to indoor modalities like an unstable surface (sand), variable environmental elements (e.g., wind, temperature), reduced gameplay and players that condition decision-making, and physical and physiological demands on athletes [8]. When compared with indoor modalities, beach sports involved a lower external workload (total distance and at lower speed, changes of speed, and impacts) but produced a higher internal workload (heart rate and rate of perceived exertion) [14,19]. Movements in sand require high levels of strength and speed due to the reduction in the applied energy in each instance of ground-to-ground contact [20]. Therefore, beach sports are demanding activities, with numerous moderate-to-high-intensity displacements and actions that are distributed intermittently throughout the game with less intense periods to facilitate recovery [15,21].

While extensive research has profiled the workload demands of invasion team sports, the literature focusing specifically on beach sports disciplines remains comparatively limited [8]. Yet, quantifying the precise external and internal loads imposed on beach athletes can, in turn, enable the individualization of training prescription and recovery, thereby, ultimately, enhancing performance and preventing overuse injuries [13]. Incorporating new technologies could enhance the analysis granularity beyond traditional monitoring approaches [7]. Therefore, the aims of this systematic review are threefold: (a) to characterize the internal and external workload demands of invasion beach sports based on competition level, age group, and sex; (b) to identify the different technologies and specific variables utilized to quantify internal and external loads in beach sports research; and (c) to report and compare the intensity zones that have been established for the various internal and external load metrics registered in beach athletes. Findings will highlight monitoring best practices to inform individualized beach training design.

## 2. Materials and Methods

### 2.1. Study Design

This manuscript is a systematic review [22] of scientific articles related to the analysis of internal and external load in invasion beach sports. The methodological procedures outlined in the Preferred Reporting Items for Systematic Reviews and Meta-Analyses (PRISMA) guidelines were followed for the development of this systematic review [23], as well as the standards for conducting systematic reviews in sports sciences [24].

### 2.2. Search Strategy and Study Eligibility

The following databases were used to search for relevant publications on 25 May 2024, after completing the registry protocol: Web of Science (Web of Science Core Collection, MEDLINE, Current Contents Connect, Derwent Innovations Index, KCI-Korean Journal Database, Russian Science Citation Index, and Scielo Citation Index), PubMed Electronics, and Scopus Electronic. The search strategy utilized to identify relevant studies with topics related to the study aims in the title, abstract, or keywords was: (“beach”) AND (“sport” OR “sports” OR “physical activity”) AND (“local positioning system” OR “LPS” OR “ultra-wideband” OR “UWB” OR “global positioning system” OR “GPS” OR “global navigation satellite system” OR “GNSS” OR “wearable” OR “inertial measurement units” OR “IMUs”) AND (“demands” OR “training load” OR “match” OR “energy expenditure” OR “internal load” OR “external load” OR “heart rate” OR “player load”).

An author (P.V.-B.) performed an electronic search to identify potentially eligible studies for this systematic review, and extracted data in an unblended, standardized manner. Then, two authors (P.V.-B and C.D.G.-C.) independently reviewed the titles, abstracts, and reference lists of retrieved studies to identify potentially relevant papers. Additionally, they evaluated the full texts of included articles to confirm those meeting the predetermined eligibility criteria. Disagreements regarding study eligibility were resolved by discussion and consensus between the two reviewers, with arbitration by a third author (J.P.-O.) when needed to resolve.

Finally, study eligibility was based on the PICOS framework as per the PRISMA guidelines [23]. The “Comparison” (C) and “Study design” (S) parameters were not considered for the inclusion/exclusion criteria as they were not critical for this systematic review. Studies were excluded if the type of document was case studies, doctoral thesis, books or book chapters, conference papers, patents, or reviews. Table 1 shows the eligibility criteria.

### 2.3. Data Extraction and Analysed Variables

The Cochrane Consumers and Communication Review Group data extraction protocol [25] was utilized to extract the following information from studies analyzing internal and external load in beach sports: (1) authors, (2) publication year, (3) sport, (4) competition level, (5) sample characteristics, (6) instruments, (7) internal and external workload variables, (8) intensity zones, and (9) referential values.

Data extraction from the included studies was performed independently by two researchers to minimize bias and error. One researcher extracted the relevant data, and the second researcher independently checked the extracted information for accuracy and completeness. Any disagreements between the two reviewers regarding the extracted data were resolved through discussion and consensus. The search results were exported as a comma-separated values (CSV) file using Windows 10 operating system. The exported data were then organized into a Microsoft Excel spreadsheet (Microsoft Corporation, Redmond, WA, USA) to systematically categorize the identified studies.

### 2.4. Quality of the Studies

The methodological quality of the included studies was evaluated using the Methodological Index for Non-Randomized Studies (MINORS) [26], which is a widely accepted and validated assessment tool for non-randomized studies. It includes 8 items for non-comparative studies and 4 additional items for comparative studies. The eight items for non-comparative studies are: (1) clearly stated aim, (2) inclusion of consecutive patients, (3) prospective data collection, (4) endpoints appropriate to study aim, (5) unbiased assessment of study endpoint, (6) follow-up period appropriate to study aim, (7) <5% lost to follow-up, and (8) prospective calculation of study size. The four additional items for comparative studies are: (9) adequate control group, (10) contemporary groups, (11) baseline equivalence of groups, and (12) adequate statistical analyses. Each item is scored as (0) not reported, (1) reported but inadequate, or (2) reported and adequate, obtaining a maximum score of 16 points for non-comparative and 24 points for comparative study designs. The MINORS quality assessment was realized by two reviewers independently, and interrater reliability was assessed by the intraclass correlation coefficient.

## 3. Results

### 3.1. Search Results

**Figure 1:**
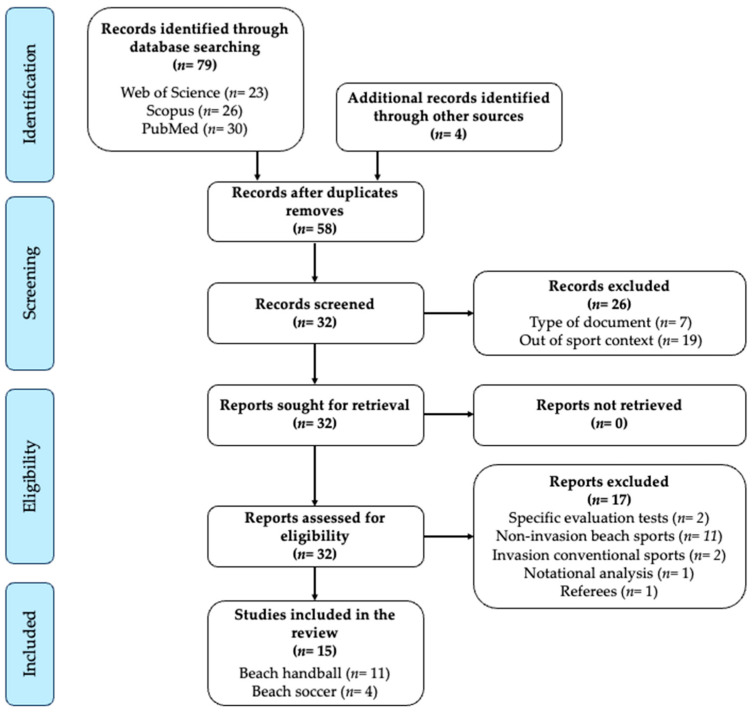
PRISMA flow diagram of study selection process.

Seventy-nine studies were identified from the database search on Web of Science (*n* = 23), Scopus (*n* = 26), and PubMed (*n* = 30). In addition, four additional studies were identified through the list of references and other sources, being a total of 83 articles. The Zotero reference manager software (version 6, Corporation for Digital Scholarship, Vienna, VI, USA) was used to import and eliminate any duplicates (25 studies). Then, 26 records were excluded from screening due to the type of document (four books or book chapters, one patent, and two conference papers) and being out of the sport context (*n* = 19). From the remaining 32 studies, 12 did not fulfill the inclusion criteria after the revision of the full text due to: (a) specific evaluation tests (*n* = 2), (b) match or training demands in non-invasion beach sports (*n* = 11), (c) match or training demands in conventional sports (n= 2), (d) the notational analysis of matches (*n* = 1), and (e) referees (*n* = 1). Finally, 15 studies that evaluate the internal and external workload in beach invasion sports were included in this systematic review: (a) beach handball (*n* = 10) [15,19,27,28,29,30,31,32,33,34,35] and (b) beach soccer (*n* = 4) [14,21,36,37]. None of the studies assessed beach rugby. A detailed representation of the selection process is illustrated in the flow diagram depicted in Figure 1.

### 3.2. Quality of the Studies

In order to evaluate the quality of the selected studies, the MINORS scale was employed [26]. Prior to the quality assessment, an inter-coder reliability analysis was conducted, yielding a value of 0.95, indicating a high level of agreement between observers (95% confidence interval: 0.93 to 0.97). The principal findings of the quality indicators for the chosen studies were as follows: (1) all studies obtained a B score with an average methodological quality of 12.93/16 (80.83%); (2) one study attained 15/16 points [15]; (3) two studies obtained 14/16 points [14,30], (4) seven studies obtained 13/16 points [19,21,27,32,33,34,37], (4) five studies achieved 12/16 points [28,29,31,35,36], and (5) no study received a score below 12 points that correspond to the C score (insufficient methodological quality) (see Table 2 for more details).

Four key aspects were primarily associated with methodological deficiencies in the selected studies: (1) Criterion 8, where 100% of studies did not report appropriately the prospective calculation of study size; (2) Criterion 2, where 73.3% of articles did not clearly acknowledge the inclusion of consecutive patients; (3) Criterion 6, where 60.0% did not report appropriately the follow-up period to study aim; and (4) Criterion 7, where 40.0% did not clearly report the <5% lost to follow-up.

### 3.3. Research Evolution, Competition Level, and Characterization of Beach Sports Athletes

Table 3 shows the research evolution (authors and year of publication), competition-level athletes’ characterization, internal and external load variables registered, and tools per beach sport. Publication dates ranged from 2010 to 2023, indicating increasing research attention on these sports from 2020 to the present (studies < 2020: *n* = 5; studies ≥ 2020: *n* = 10). The included studies examined beach soccer and beach handball players ranging from amateur to professional international levels. In beach soccer, athletes competed at the amateur [37] and national level [14,21,36]. For beach handball, regional- [28,35], national- [15,29], European- [33,34], and international-level players [19,27,30,31,32] were analyzed. This indicates the research has progressed from recreational to elite settings.

The beach athletes present the following characteristics: (a) age—in beach soccer, ranging from 23.6 ± 4.4 to 29.4 ± 6.9 years, and, in beach handball, ranging from 20.1 ± 4.9 to 26.3 ± 4.8 years; (b) height—in beach soccer, ranging from 1.82 ± 0.06 to 1.77 ± 0.05 m, and, in beach handball, ranging from 1.87 ± 0.09 to 1.78 ± 0.04 m; and (c) body mass—in beach soccer, ranging from 79.3 ± 9.1 to 71.8 ± 3.8 kg, and, in beach handball, ranging from 86.9 ± 9.5 to 77.6 ± 13.4 kg. Beach handball players were younger, taller, and heavier than beach soccer players. Regarding sex differences in beach handball, male players were taller (male vs. female: 1.87–1.78 vs. 1.70–1.66 m) and heavier (male vs. female: 86.9–77.6 vs. 70.5–60.0 kg) than female players.

A variety of technologies were used, primarily GPS, UWB systems, accelerometers, and heart rate monitors. Earlier works relied more on video analysis (*n* = 1) [37], while recent studies integrated EPTS units (*n* = 13; e.g., WIMU PRO, SPI Pro X, Optimeye S5) to capture the external load [14,15,19,21,27,28,29,30,31,32,33,34,35]. The internal load assessment has been carried out via a heart rate band (*n* = 11; Polar Electro and coded T14 systems) [15,19,21,27,29,30,32,33,34,36,37], a lactate meter (*n* = 2) [36,37] or psychological rated effort (*n* = 4) [14,30,33,34].

External load variables focused on locomotive demands like total distance or per different speed zones (*n* = 10) [14,15,19,21,27,29,31,32,33,34], accelerations/decelerations or per intensities (*n* = 7) [15,19,27,30,31,32,33,34], jumps (*n* = 7) [15,28,30,31,33,34,35], impacts or per intensities (*n* = 5) [15,19,28,31,35], steps (*n* = 3) [15,28,35], changes of direction (*n* = 2) [30,31], player load (*n* = 7) [15,28,30,31,33,34,35], or body load (*n* = 2) [19,29]. For the internal load, average, maximum, and minimum HR (*n* = 10) continue to be primary measures [15,19,21,29,32,33,34,36,37], supplemented by time in HR zones (*n* = 5) [19,21,27,32,33], blood lactate (*n* = 2) [36,37], RPE (*n* = 2) [14,30], and TRIMP (*n* = 2) [33,34] more recently.

### 3.4. External Workload Demands and Intensity Zones

The external workload demands and intensity zones profile in beach sports players are showed in Table 4. Players covered 929.5 ± 269.5 m (566-to-1606 m.) [14,15,19,27,29,31,32,33,34] at an average speed of 3.1 ± 0.95 km/h (2.5-to-4.2 km/h) [19,29,32], and reached a maximum speed of 16.5 ± 3.3 km/h (11.9-to-21.7 km/h) [15,21,27,29,31,32,33,34]. Athletes performed 368.2 ± 103.4 (268-to-533) accelerations over 0 m/s^2^ [15,34,35] or 41.1 ± 13.3 (19-to-53) accelerations over 1 m/s^2^ [19,27,30,31,33], and suffered 718-to-1251 impacts over 0 g [29], 477-to-572 impacts over 2 g [28] or 78-to-95 impacts over 5 g [19]. In addition, beach players realized 8.3 ± 4.1 (4-to-14) jumps [28,30,31,33,34,35] and 815.3 ± 45.1 (765-to-852) steps [28,35] that entail a player load of 12.5 ± 2.9 a.u. (8.8-to-16.2 a.u.) by RealTrack Systems or 125.1 ± 29.7 a.u. (88-to-162 a.u.) by Catapult Sports [15,28,30,31,33,34,35] and a body load of 18.8 ± 5.9 a.u (11.3-to-24.4) [19,29].

Intensity zones vary according to selected studies in distance, accelerations, and impacts. Regarding the distance covered, two procedures to fix intensity zones were identified: (1) video analysis with five zones (standing, walking, jogging, running, and sprinting) considering the intensity of movements by coders [37], and (2) tracking technologies (GPS or UWB) with five or six zones considering the speed of displacements [14,15,19,21,27,32,33,34]. With five intensity zones, two classifications were found: (1) Z1 0–4 km/h, Z2 4–7 km/h, Z3 7–13 km/h, Z4 13–18 km/h, and Z5 >18 km/h [21]; and (2) Z1 1–5.9 km/h, Z2 6–8.9 km/h, Z3 9–11.9 km/h, Z4 12–14.9 km/h, and Z5 >15 km/h [33,34]. In addition, three studies modified the classification utilized by Castellano and Casamichana [21] with six intensity zones dividing the lowest intensity zone into two zones: standing (0–0.4 km/h) and walking (0.4–4 km/h) [15,19,32]. Finally, Lara-Cobos et al. [27] utilized a different classification system based on the maximum speed of the session dividing the distance covered in six zones: Z1 (<10% S_max_), Z2 (10–29% S_max_), Z3 (30–49% S_max_), Z4 (50–79% S_max_), Z5 (80–95% S_max_), and Z6 (>95% S_max_). The majority of displacements (85–90%) were realized <13 km/h [14,15,19,21,27,32,33,34].

Accelerations were classified in three zones (Z1: 1–2 m/s^2^, Z2: 2–3 m/s^2^, and Z3: >3 m/s^2^) [19,27] or four zones, adding a highest intensity zone (Z4: >4 m/s^2^) [15]. One study also counted only accelerations over 2,5 m/s^2^ [34]. Almost all acceleration were performed >2 m/s^2^ [15,19,27]. On the other hand, three different settings of impact intensity zones were found: (1) three zones (0–5 g, 5–8 g, and >8 g) [35], (2) five zones (2–4 g, 4–6 g, 6–8 g, 8–10 g, and >10 g) [28], or (3) six zones (5–6 g, 6–6.5 g, 6.5–7 g, 7–8 g, 8–10 g, and >10 g) [19]. The greatest number of impacts suffered are of low or very low intensity (<6 g) [19,28,35].

When comparing beach sports, beach soccer covered a greater total distance (1370 vs. 860 m) and a greater distance over 13 km/h (140 vs. 45 m) [14,15,19,27,29,31,32,33,34], and achieved a higher maximum speed (21.7 vs. 15.9 km/h) [15,21,27,29,31,32,33,34]. No data were available to compare the accelerations, impacts, jumps, steps, player load, or body load between beach handball and beach soccer. In terms of sex-related external workload during beach handball, male players covered a greater total distance (+200–300 m) and a greater distance over 13 km/h (+15–25 m), reached a higher maximum speed (+2–3 km/h), performed more total accelerations (+30–60), and experienced more impacts (+100–300). These higher external workload demands resulted in a higher player load (2–4 a.u. calculated by RealTrack; +20–40 a.u. calculated by Catapult Sports) and body load (+3–4 a.u.) [15,19,28,29,33,34].

### 3.5. Internal Workload Demands and Intensity Zones

Table 5 represents the internal workload demands and intensity zones profile in beach sports players. Athletes demonstrated an average heart rate between 137–170 bpm, a minimum heart rate between 117–129 bpm, and a maximum heart rate between 166–192 bpm [15,19,21,29,32,33,34,36,37]. During the intermittent activity profile of beach sports, players developed anaerobic and aerobic fitness due to the heart rate demands ranging from 60% to 95% of the maximum heart rate [19,21,32,33,34,37].

Heart rate zones were classified according to the percentage of the maximum heart rate (%HRmax) in four zones (Z1: <75%, Z2: 76–84%, Z3: 85–89%, and Z4: >90%) [21], five zones (Z1: <65%, Z2: 66–75%, Z3: 76–85%, Z4: 86–95%, and Z5: >95%) [37], or six zones (Z1: <60%, Z2: 60–70%, Z3: 70–80%, Z4: 80–90%, Z5: 90–95%, and Z6: >95%) [15,19,27,32,33,34]. In addition, players performed a TRIMPS of 57.8-to-69.4 a.u. or a sHRZ of 77.4 ± 26.5, and reported a Hooper index of 8/27 a.u. and a RPE of 6–8/10 [14,30,33,34]. Finally, 6.7 to 7.0 mmoL of blood lactate have been registered during beach sports [36,37].

When comparing beach handball and beach soccer, both modalities produced similar heart rate demands (average, maximum, minimum, and intensity zones) and rate of perceived exertion. In the same way, no sex-related differences were found on internal workload demands between male and female handball players [15,19,28,29,33,34].

## 4. Discussion

Participation in beach sports has increased over the last decade. However, the research characterizing the workload demands in beach sports is comparatively limited compared to indoor modalities. Precisely quantifying external and internal loads in training and competition enables the individualization of training prescription and recovery for performance optimization and injury prevention. Therefore, this systematic review aimed to: (a) characterize the workload demands of invasion beach sports, (b) identify technologies and variables utilized to quantify loads, and (c) report intensity zones for load metrics.

### 4.1. External Workload Demands

Beach soccer and beach handball, as beach invasion sports, present unique external load demands due to their specific playing conditions. In beach soccer, players cover more total distance (1606 ± 88 m) and achieve higher maximum speeds (21.7 ± 4.5 km/h), as well as more meters in high-speed zones (110 ± 38 in Z4, 30 ± 28 in Z5) [14,21]. Beach handball, on the other hand, involves more explosive and high-intensity actions (533 ± 309 accelerations), including rapid accelerations, decelerations, frequent jumps (12 ± 5), and a higher impact (117 ± 60 in Z3, 25 ± 13 in Z4) [33,35]. These differences can be explained by factors such as: court size, sports rules, or game dynamics [38,39,40]. Concerning the court size, beach soccer presents a bigger playing area, allowing soccer players to cover longer distances, reaching higher speeds more frequently [38]. In relation to sport rules, beach handball players can be substituted several times during the game, reducing the volume load values and increasing potential explosive efforts [39]. Furthermore, the unique dynamics of beach handball involve a predominant stationary attack vs. defense interaction near the goal area, where explosive movements such as changes in direction, jumps, and “in-flight” acrobatic moves are necessary in order to generate scoring opportunities [39,40]. As a result, beach handball players experience a greater number of accelerations and decelerations, as well as frequent jumps and impacts in comparison to beach soccer.

### 4.2. Internal Workload Demands

Regarding the internal workload, both beach soccer and beach handball players display significant cardiovascular demands, as shown by their average heart rates ranging from 137–170 bpm [19,32]. The minimum and maximum values also reflect the intense nature of these sports, ranging from 117–129 bpm to 164–192 bpm, respectively [32,34,37]. Moreover, players can reach 20% of total game time in the maximum heart rate zone (95% HR_max_), indicating the considerable demands imposed on the cardio-vascular system during the match [32,37]. Moreover, metrics like RPE and blood lactate can also assess the internal load in beach invasion sports. RPE scores typically range from 6 to 8 out of 10, indicating a high level of exertion experienced by players during matches [14,30]. Concurrently, blood lactate concentrations ranging from 6.7 to 7.0 mmol/L underscore the metabolic stress and anaerobic energy system [36,37]. In addition, the internal load demands in beach soccer and beach handball have direct implications for training and performance optimization. The substantial time spent in higher heart rate zones requires targeted endurance and high-intensity interval training. Moreover, the high RPE and lactate levels suggest the need for recovery strategies and metabolic conditioning. Understanding these internal load metrics is essential in order for coaches and technical staffs to design specific training sessions that enhance performance while minimizing the risk of overtraining and injury. This approach ensures that players achieve the physiological demands of match-play in beach invasion sports, leading to optimal performance outcomes.

### 4.3. Intensity Zones

Determining performance intensities zones based on internal and external load variables can be challenging due to different methodologies and technologies used. Various devices and brands, and the author’s decision to divide the reference intensity zones considering a proposed criterion lead to variability. This variability makes it difficult to compare data across studies and sports [21,27,34,37]. Reference studies have highlighted the need for individualized intensity reference zones based on athletes characteristics for better player load management [41,42]. However, a common criterion for establishing intensity zones in beach sports would facilitate data integration and interpretation [43,44]. For this purpose, the evaluation of raw data of the magnitude of speeds, accelerations, decelerations, and impacts, and the classification by Gaussian distributions, k-means clustering, or spectral clustering could provide objective intensity zones to classify external workload demands [45,46]. From these objective zones, a unified approach would help in comparing and generalizing across studies, which can be useful in developing sport-specific training and recovery protocols. Therefore, it is necessary to carry out extensive methodological studies led by experts to establish reference intensity zones for internal and external load variables according to each sport and playing context’s requirements.

### 4.4. Sex-Related Differences in Beach Sports

Male players exhibit a higher external workload in beach handball compared to female players. This is characterized by a greater total distance covered (additional 200–300 m), a greater distance over 13 km/h (extra 15–25 m), a higher maximum speed (additional 2–3 km/h), increased total accelerations (additional 30–60), and more impacts (additional 100–300) [15,19,28,29,33,34]. These differences in external workload can be explained by considering differences in physical capacities, strength, and types of effort exerted during the game. Male beach handball players have higher values of weight, body mass index, anthropometric characteristics, and strength, that allow them to move larger distances and at a higher intensity during competition [19,28,34].

Moreover, despite the pronounced differences in external workload, studies indicate no significant sex-related differences in internal workload demands between male and female handball players [19,33,34]. This suggests that, although male players may engage in a more intense external load, the internal physiological response and exertion experienced by female players are comparable. Factors such as heart rate, perceived exertion, and metabolic stress do not show marked differences between sexes, indicating that females experience the same internal load at lower external demands in beach sports.

### 4.5. Workload Demands for Indoor vs. Beach Sports

Comparative studies have found that indoor sports like handball and soccer require higher external workload demands, while beach sports require a significantly higher internal workload [19,21,47,48]. This difference is mainly attributed to the nature of the playing surfaces [49]. Beach sports, with their softer and unstable sand surface, require more energy expenditure and muscle engagement for movement, which increases the internal load despite lower external load metrics like the total distance covered and speed [19,21,47,48]. The unstable and yielding nature of the beach surface plays a crucial role in influencing the demands of beach sports [40]. Activities like running, jumping, and changing direction are more physically demanding on sand compared to the solid surface of indoor courts [49]. Additionally, variable outdoor conditions such as wind and temperature further contribute to the increased physical demands on beach sports athletes [49]. Findings suggest that training in beach conditions, characterized by challenging environments like sand, significantly impacts an athlete’s aerobic capacity and running economy [50]. The distinct workload profiles of indoor and beach sports require custom training approaches for athletes. For beach sports, training should emphasize developing the strength and endurance required to overcome the challenges of the sand surface and variable conditions. In contrast, indoor sports training would focus on repeated speed and agility exercises, to better adapt to the higher external load demands of these sports [47,48]. Understanding these workload demands is crucial for optimizing athletic performance and formulating effective injury prevention strategies [40,49].

### 4.6. Considerations on Electronic Performance and Tracking Systems (EPTSs) in Beach Sports

The use of EPTSs in beach sports requires the careful consideration of various factors that can influence the accuracy, reliability, and validity of the collected data. These factors include tracking positioning, microtechnology data accuracy, device attachment and comfort, and data transmission methods.

*Tracking, Microtechnology, and Precision:* EPTS devices are composed of tracking technologies such as GPS in outdoor environments and LPS in indoor environments to register player movements and positions during beach sports matches and training sessions. However, the accuracy and precision of these systems can be affected by several factors, including satellite availability, environmental conditions (e.g., wind, temperature, and humidity), and the presence of obstacles or obstructions in the playing area [51,52]. In beach sports, the open and unobstructed playing environment may provide better satellite visibility and signal reception, potentially improving the accuracy of GPS-based tracking systems. However, the presence of sand and other environmental factors may introduce signal interference or multipath effects, which can degrade the positioning precision [51,52].

In addition to tracking systems, EPTSs often incorporate microtechnologies such as accelerometers, gyroscopes, and magnetometers to capture high-intensity actions like jumps, changes in direction, and impacts [11,53]. The precision of these microtechnologies can be influenced by factors such as sensor quality, sampling rates, and data processing algorithms [11,53]. It is essential that we consider the specifications and limitations of the microtechnologies used in EPTSs to ensure the accurate and reliable measurement of these high-intensity actions, which are particularly relevant in beach sports due to different movements without displacement (e.g., vertical jumps and impacts).

*Device Attachment, Comfort, and Validity:* The attachment of EPTS devices to the athletes’ body is commonly realized by a customized vest at the interscapular level. Its attachment is a critical factor that can influence the comfort and precision of the collected data [53]. In beach sports, athletes typically wear minimal clothing which can pose challenges in securely attaching EPTS devices while maintaining athlete comfort and minimizing potential movement artifacts. Additionally, the presence of sand and sweat may affect the adhesion and stability of the devices, especially of heart rate bands, potentially compromising data validity [54]. Researchers and practitioners should carefully consider the attachment methods, device placement, and athlete feedback to ensure optimal data collection while maintaining athlete comfort and performance.

*Data Transmission and Real-Time Monitoring:* The transmission of data from EPTS devices to external systems is another important aspect to consider in beach sports. Real-time monitoring and data transmission can provide valuable insights for coaches and support staff during matches or training sessions, enabling them to make informed decisions and adjustments [55]. However, the beach environment may introduce challenges in wireless data transmission, potentially affecting the reliability and latency of real-time data streaming [56]. Alternative methods, such as post-session data retrieval, may be necessary in certain situations, which could impact the ability to make immediate adjustments during the activity.

To address these considerations, researchers and practitioners should carefully evaluate the capabilities and limitations of the EPTSs used in beach sports, and consider the specific environmental and practical challenges associated with these sports. Collaboration between sports scientists, engineers, and manufacturers is essential in order to develop tailored solutions that ensure accurate and reliable data collection while maintaining athlete comfort and performance. Additionally, conducting validation and reliability studies in beach sports settings can provide valuable insights and inform best practices for the effective use of EPTSs in these unique environments.

### 4.7. Limitations and Future Research

While this systematic review provides valuable insights into the workload demands of beach invasion sports, some limitations should be acknowledged based on the included studies. The majority of research has focused on beach handball, with relatively fewer studies examining beach soccer. This limits the ability for us to draw comprehensive comparisons between the two sports. Additionally, most studies analyzed elite or professional athletes, leaving a gap in the understanding of the demands across different competitive levels, such as amateur or recreational participants. Furthermore, several studies did not report detailed information on participant characteristics, such as years of experience or playing position, which could influence the interpretation of workload profiles. Lastly, the variability in technologies used and the lack of standardized intensity zones for external load metrics hindered direct comparisons across studies and sports, highlighting the need for consistent methodological approaches in future research.

Standardizing methodological approaches is a pressing need for future research in beach sports. Establishing consensus on intensity zones for external load metrics, such as the distance covered, accelerations, and impacts, would facilitate meaningful comparisons across studies and sports. Additionally, efforts should be made to integrate emerging wearable technologies that can comprehensively assess internal workload demands. In particular, incorporating breathing pattern monitoring could complement the traditionally used measures of heart rate and lactate, providing a more holistic understanding of the physiological strain experienced by athletes during beach sports competitions and training.

Furthermore, investigations should explore the potential influence of environmental factors, such as wind, temperature, and humidity, on the workload demands of beach sports. These unique conditions may significantly impact both external and internal loads, necessitating tailored training and recovery strategies. Research should also examine potential differences in workload profiles based on playing positions, enabling the development of position-specific training protocols for optimal performance and injury prevention. Expanding the scope of research to include a broader range of competition levels, from recreational to elite, would provide a more comprehensive understanding of the sport-specific demands and inform appropriate training programs across various athlete populations.

## 5. Conclusions

This systematic review characterized the internal and external workload demands of beach invasion sports across competition levels, ages, and sexes. Key findings demonstrate beach sports are intermittent in nature, entailing moderate-to-high-intensity bouts interspersed with lower-intensity activity for recovery. Average and peak heart rates align with high-intensity interval training principles for both aerobic and anaerobic adaptations. The unstable sand surface and variable outdoor conditions elicit a greater perceptual effort despite lower external load volumes versus indoor equivalents. Male players showed higher locomotive demands, but no internal workload differences exist between sexes. Finally, acceleration, impact, and internal load intensity zones vary substantially in the limited beach sports research, highlighting an urgent need for standardization to enable comparisons and inform training design.

### Practical Applications

The data on internal and external workload demands in beach sports can be leveraged for individualized training prescription and recovery through several approaches. Firstly, by integrating the external load metrics (e.g., distances covered, accelerations, and impacts) with individual athlete characteristics such as age, sex, playing position, and training age, coaches can tailor training drills and intensities to optimally prepare each player for the specific demands of competition. Secondly, the internal load data, particularly the heart rate responses and perceived exertion, can be used to monitor individual athletes’ physiological and psychological readiness, enabling appropriate adjustments to training loads and recovery strategies.

For this purpose, standardized external load zones are needed, while internal load zones by percentage of maximal heart rate appear reasonably consistent. Coaches should recognize that an unstable surface accentuates internal stress, enabling high cardio respiratory training stimuli despite deceptively lower running volumes. Training should prepare players physically and perceptually for intermittent effort profiles. Finally, male and female beach players likely require similar cardio conditioning, but more position-specific locomotive development.

Moreover, the integration of EPTSs with athlete monitoring systems can facilitate real-time feedback and online monitoring processes. Through real-time data, adjustments can be made during training sessions or matches based on individual athletes’ external and internal load responses. This real-time monitoring can help prevent excessive fatigue, overload, or underload, ultimately reducing the risk of injury and optimizing performance. Furthermore, by combining the workload data with additional personal biological parameters, such as hormonal profiles, sleep quality, and nutrient intake, a more comprehensive understanding of each athlete’s readiness and recovery status can be achieved. This holistic approach can inform personalized periodization strategies, tailoring training phases, intensities, and recovery interventions to individual needs.

## Figures and Tables

**Table 1:** Eligibility criteria.

PICOS	Search Words	Inclusion Criteria	Exclusion Criteria
Population	Beach, sport	Athletes that participate on invasion beach sports.	Athletes that participate in conventional sports or other non-invasion beach sports.
Intervention/ Exposure	Local Positioning System or LPS, Ultra-Wide Band or UWB, Global Positioning System or GPS, Global Navigation Satellite Systems or GNSS, Wearable, Inertial Measurement Units or IMUs.	Using one of the non-invasive and portable technologies for monitoring internal or external workload.	Not using one of the non-invasive and portable technologies for monitoring internal or external workload.
Outcomes	Demands, Training Load, Match Performance, Energy Expenditure, Internal Load, External Load, Heart Rate, or Player Load.	Studies should refer to load monitoring (internal or external load) and register specific variables in training and competition contexts.	Studies that did not register internal and external workload variables, and realize non-ecological assessments out of the training and competition contexts.

**Table 2:** Methodological quality of selected studies.

Selected Studies (Authors and Year)	MINORS Criteria								Total Score
	1	2	3	4	5	6	7	8	
Castellano and Casamichana (2010) [21]	2	1	2	2	2	1	2	1	13/16
Scarfone et al. (2015) [37]	2	2	2	2	1	1	2	1	13/16
Bozdogan (2017) [36]	2	1	2	2	1	2	1	1	12/16
Pueo et al. (2017) [19]	2	1	2	2	1	2	2	1	14/16
Gutiérrez-Vargas et al. (2019) [29]	2	1	2	2	2	1	1	1	12/16
Gómez-Carmona et al. (2020) [28]	2	1	2	2	2	1	1	1	12/16
Mancha-Triguero et al. (2020) [35]	2	1	2	2	2	1	1	1	12/16
Zapardiel and Asín-Izquierdo (2020) [34]	2	2	2	2	2	1	1	1	13/16
Iannaccone et al. (2021) [30]	2	2	2	2	1	2	2	1	14/16
Sánchez-Sáez et al. (2021) [32]	2	1	2	2	1	2	2	1	13/16
Costa et al. (2022) [14]	2	1	2	2	2	2	2	1	14/16
Müller et al. (2022) [31]	2	1	2	2	2	1	1	1	12/16
Gómez-Carmona et al. (2023) [15]	2	2	2	2	2	2	2	1	15/16
Cobos et al. (2023) [27]	2	1	2	2	2	1	2	1	13/16
Zapardiel et al. (2023) [33]	2	1	2	2	2	1	2	1	13/16

**Note.** MINORS criteria: (1) clearly stated aim, (2) inclusion of consecutive patients, (3) prospective data collection, (4) endpoints appropriate to study aim, (5) unbiased assessment of study endpoint, (6) follow-up period appropriate to study aim, (7) <5% lost to follow-up, and (8) prospective calculation of study size.

**Table 3:** Research evolution, competition level, athletes’ characteristics, assessment instruments, and registered variables per beach sport.

Selected Studies (Authors and Year)	Players’ Level	Athletes’ Characteristics					Instruments		Variables	
		Nº	Gender	Age (Years)	Height (Meters)	Body Mass (kg)	External Load Tool, Technology (Company)	Internal Load Tool, Variable (Company)	External Load	Internal Load
***Beach soccer***										
Castellano and Casamichana (2010) [21]	National (Spanish League)	10	male	25.5 ± 0.5	1.80 ± 0.08	78.2 ± 5.6	MinimaxX 2.0, GPS (Catapult Sports)	Team Sport, HR (Polar)	TD and per speed zones, Speed_max_	HR_avg_, HR_min_, HR_max_ and %HR_max_ per zones
Scarfone et al. (2015) [37]	Amateur (3 years’ experience)	10	male	23.6 ± 4.4	1.77 ± 0.05	71.8 ± 3.8	Hi8 Pro Camera, Video (SONY)	Team Sport, HR (Polar) Accusport, Lactate (Roche)	% time per locomotive category	HR_avg_, HR_max_, HR_min_ Blood lactate
Bozdogan (2017) [36]	National (Turkish League)	12	male	28.33 ± 3.70	1.79 ± 0.08	79.3 ± 9.1		No model, HR (Polar) No model, Lactate (Scout)		HR_avg_, HR_max_ Blood lactate
Costa et al. (2022) [14]	International (Portugal National Team)	11	male	29.4 ± 6.9	1.82 ± 0.06	74.1 ± 7.9	Apex, GPS (STATSports)	Hooper Index, RPE	TD and per speed zones	Hooper Index
***Beach handball***										
Pueo et al. (2017) [19]	International (Spanish National Team)	12 12	male female	26.3 ± 4.8 23.7 ± 4.8	1.87 ± 0.09 1.68 ± 0.05	84.5 ± 12.1 62.4 ± 4.6	SPI Pro X, GPS (GPSport)	Electro, HR (Polar)	TD and per speed zones, Speed_avg_, Imp, Acc per intensities, BL	HR_avg_, HR_max_ and %HR_max_ per zones
Gutiérrez-Vargas et al. (2019) [29]	National (Costa Rican Tournament)	8 8	male female	25.6 ± 9.0 26.0 ± 7.0	1.78 ± 0.04 1.67 ± 0.08	78.1 ± 6.5 70.5 ± 12.7	SPI Pro X II, GPS (GPSport)	Coded T14, HR (Polar)	TD, Speed_avg_, Speed_max_, BL, Imp	HR_avg_
Gómez-Carmona et al. (2020) [28]	Regional (Senior Extremadura Championship)	20 25	male female	23.92 ± 0.05 19.54 ± 3.72	1.79 ± 0.08 1.66 ± 0.06	80.71 ± 13.83 58.65 ± 8.46	WIMU PRO, Acel (RealTrack)		Total Imp and per intensities, PL, steps, jumps	
Mancha-Triguero et al. (2020) [35]	Regional (U-16 Extremadura Championship)	20	male	15.6 ± 0.3	1.72 ± 0.06	65.52 ± 9.94	WIMU PRO, Acel (RealTrack)		Total Imp and per intensities, Acc/Dec, PL, steps, jumps	
Zapardiel and Asín-Izquierdo (2020) [34]	International (European Championship 2017)	25 32	male female	25.3 ± 4.8 25.3 ± 4.8	1.87 ± 0.07 1.68 ± 0.04	86.9 ± 9.5 60.7 ± 3.8	OptimEye S5, GPS (Catapult)		TD, Acc/Dec, Jumps, PL	HR_avg_, TRIMPS
Iannaccone et al. (2021) [30]	International (U17 Lithuanian National Team)	13	male	15.9 ± 0.3	1.80 ± 0.10	67.4 ± 6.8	Clearsky, GPS (Catapult)	H10 Electro, HR (Polar)	PL, Acc/Dec, CoD, Jumps, Events per intensities	SHRZ score, RPE
Sánchez-Sáez et al. (2021) [32]	International (Spanish National Team)	9	female	24.6 ± 4.0	1.68 ± 0.06	62.4 ± 4.6	SPI Pro X, GPS (GPSport)	Electro, HR (Polar)	TD and per speed zones, Speed_avg_, Speed_max_	HR_avg_, HR_max_ and %HR_max_ per zones
Müller et al. (2022) [31]	International (German National Team)	69	male	20.1 ± 4.9	1.87 ± 0.09	83.9 ± 11.5	Clearsky, GPS (Catapult)		TD, Speed_max_, Max Acc/Dec, Total Imp, EE, PL, CoD, Jumps	
Gómez-Carmona et al. (2023) [15]	National (Brazilian League)	54 38	male female	22.1 ± 2.6 24.4 ± 5.5	1.80 ± 0.05 1.70 ± 0.05	77.6 ± 13.4 67.5 ± 6.5	WIMU PRO, UWB (RealTrack)	No model, HR (Garmin)	TD and per speed zones, Speed_max_, Total Acc/Dec and per intensities, Total Imp and per intensities, PL, steps, jumps	HR_avg_
Cobos et al. (2023) [27]	International (Spanish National Team)	14	female	24.6 ± 4.0	1.69 ± 0.06	60.0 ± 4.1	SPI HPU, GPS (GPSport)	Electro, HR (Polar)	TD, Speed_max_, Acc/Dec per intensities	%HR_max_ per zones
Zapardiel et al. (2023) [33]	International (Europe Championship)	25 32	male female	25.38 ± 4.82 25.38 ± 4.82	1.87 ± 0.07 1.68 ± 0.04	86.96 ± 9.53 60.78 ± 3.87	OptimEye S5, GPS (Catapult)	Electro, HR (Polar)	TD and per speed zones, Speed_max_, Acc/Dec, Jumps, PL	HR_avg_, HR_max_, HR_min_, %HR_max_ per zones, TRIMPS

**Note.** N: sample; GPS: Global Positioning Systems, UWB: Ultra-Wide Band; Acel: Accelerometer; HR: Heart Rate; TD: Total Distance; Speed_max_: Maximum Speed; Speed_avg_: Average Speed; Acc: Accelerations; Dec: Decelerations; Imp: Impacts; PL: Player Load; BL: Body Load; EE: Energy Expenditure; MP: Metabolic Power; CoD: Changes of Direction; HR_AVG_: Average Heart Rate; HR_max_: Maximum Heart Rate; HR_avg_: Average Heart Rate; HR_min_: Minimum Heart Rate; TRIMP: Training Impulse.

**Table 4:** External workload demands (mean ± SD) and intensity zones (cursive text) in beach sports.

Selected Studies (Authors and Year)	Players’ Level N/Sex	Distance (Zones in km/h; e.g., *13–17.9* or *Running*) Meters/% Time ± SD						Speed (km/h)		Accelerations (Zones in m/s^2^; e.g., *2–3*) *n* ± SD					Impacts (Zones in g Force; e.g., *8–10*) *n* ± SD						Jumps *n* ± SD	Steps *n* ± SD	PL/BL a.u. ± SD
		Total	Z1	Z2	Z3	Z4	Z5	AVG	MAX	Total	Z1	Z2	Z3	Z4	Total	Z1	Z2	Z3	Z4	Z5			
***Beach soccer***																							
Castellano and Casamichana (2010) [21]	National 10/male	1135 ± 27	*0–3.9* 249 ± 25	*4–6.9* 297 ± 57	*7–12.9* 422 ± 132	*13–17.9* 110 ± 38	*>18* 30 ± 28		21.7 ± 4.5														
Scarfone et al. (2015) [37]	Amateur 10/male		*standing* 35 ± 6	*walking* 46 ± 5	*jogging* 5 ± 1	*running* 12 ± 4	*sprinting* 2 ± 1																
Bozdogan (2017) [36]	National 12/male																						
Costa et al. (2022) [14]	International 11/male	1606 ± 88				*>13* 66 ± 18																	
***Beach handball***																							
Pueo et al. (2017) [19]	International 12/male 12/female	1235 ± 192 1118 ± 222	*0–4* 398 ± 64 409 ± 65	*4.1–7* 433 ± 103 371 ± 94	*7.1–13* 356 ± 101 318 ± 109	*13.1–18* 46 ± 32 20 ± 16	*>18* 2 ± 4 0	4.2 ± 0.6 3.9 ± 0.8		53 ± 17 45 ± 16	*1–2* 43 ± 12 40 ± 13	*2–3* 9 ± 5 4 ± 3	*>3* 1 ± 1 0		78 ± 25 95 ± 29	*5–6* 40 ± 14 51 ± 15	*6–7* 21 ± 5 21 ± 6	*7–8* 6 ± 3 10 ± 4	*8–10* 4 ± 4 6 ± 4	*>10* 8 ± 4 6 ± 5			*BL* 22.7 ± 9.2 24.4 ± 9.5
Gutiérrez-Vargas et al. (2019) [29]	National 8/male 8/female	939 ± 212 613 ± 145						2.8 ± 0.6 1.8 ± 0.4	15.9 ± 2.1 13.6 ± 2.2						1251 ± 30 718 ± 138								*BL* 16.7 ± 7.4 11.3 ± 4
Gómez-Carmona et al. (2020) [28]	Regional 20/male 25/female														572 ± 266 477 ± 218	*2–4* 467 ± 175 381 ± 168	*4–6* 125 ± 105 77 ± 47	*6–8* 31 ± 20 14 ± 10	*8–10* 7 ± 5 3 ± 3	*>10* 4 ± 5 2 ± 3	6 ± 5 4 ± 3	765 ± 309 852 ± 294	*PL* 15.1 ± 5.7 14.4 ± 4.4
Mancha-Triguero et al. (2020) [35]	Regional/ 20/male									533 ± 309					476 ± 192		0–5 334 ± 130	5–8 117 ± 60	>8 25 ± 13		5 ± 3	829 ± 267	*PL* 17.4 ± 4.8
Zapardiel and Asín-Izquierdo (2020) [34]	International 25/male 32/female	891 ± 313 739 ± 317	*1–5.9* 581 ± 244 440 ± 200	*6–8.9* 133 ± 100 145 ± 89	*9–11.9* 76 ± 80 63 ± 60	*12–14.9* 18 ± 24 21 ± 25	*>15* 2 ± 2 2 ± 5		20.5 ± 4.3 18.4 ± 0.4	396 ± 110 338 ± 105		*>2.5* 9 ± 4 5 ± 6									12 ± 5 5 ± 5		*PL* 10.3 ± 3.4 8.8 ± 3.6
Iannaccone et al. (2021) [30]	International 13/male									26 ± 14											14 ± 10		*PL* 10.9 ± 4.2
Sánchez-Sáez et al. (2021) [32]	International 9/female	898 ± 216	*0–4* 485 ± 132	*4.1–7* 262 ± 77	*7.1–13* 123 ± 39	*>13.1* 27 ± 25		2.5 ± 0.6	14.9 ± 2.3														
Müller et al. (2022) [31]	International 69/male	806 ± 214							16.5 ± 2.0	19 ± 9											11 ± 7		*PL* 9.2 ± 2.8
Gómez-Carmona et al. (2023) [15]	National 54/male 38/female	754 ± 250 566 ± 252	*0–4* ND ND	*4–7* 226 ± 82 164 ± 80	*7–13* ND ND	*13–18* 81 ± 38 58 ± 30	*>18* 2 ± 10 4 ± 4		20.7 ± 8.1 15.8 ± 2.8	306 ± 72 268 ± 92	*1–2* ND ND	*2–3* ND ND	*3–4* ND ND	*>4* 40 ± 60 20 ± 20									*PL* 16.2 ± 5.1 12.7 ± 6.0
Cobos et al. (2023) [27]	International 14/female	702 ± 251	*0–4.7* 313	*4.7–8* 91	*8–12* 225	*12–14.7* 41	*>14.7* 2		15.5 ± 1.9	59 ± 37	*1–2* 36 ± 16	*2–3* 20 ± 12	*>3* 2 ± 7										
Zapardiel et al. (2023) [33]	International 25/male 32/female	1059 ± 420 882 ± 380	*1–5.9* 595 ± 241 506 ± 239	*6–8.9* 214 ± 145 204 ± 116	*9–11.9* 134 ± 116 82 ± 73	*12–14.9* 31 ± 52 36 ± 75	*>15* 3 ± 11 5 ± 13		11.9 ± 2.6 12.1 ± 2.9	47 ± 10 39 ± 13											13 ± 10 2 ± 5		*PL* 12.0 ± 4.3 10.6 ± 4.6

**Note.** N: sample; *n*: count; SD: standard deviation; PL: player load; BL: body load; AVG: average; MAX: maximum; Z1, Z2, Z3, Z4, and Z5: Zone 1, 2, 3, 4, and 5; ND: no data.

**Table 5:** Internal workload demands (mean ± SD) and intensity zones (cursive text) in beach sports.

Selected Studies (Authors and Year)	Players’ Level N/sex	HR_avg_ (bpm)	HR_min_ (bpm)	HR_max_ (bpm)	HR Zones (Zones in %HR_max_; e.g., *90–95%*) % Time ± SD						TRIMPS/sHRZ (a.u. ± SD)	Hooper Index/RPE (1–10) (a.u. ± SD)	Blood Lactate (mmol ± SD)
					Z1	Z2	Z3	Z4	Z5	Z6			
***Beach soccer***													
Castellano and Casamichana (2010) [21]	National 10/male	165 ± 20	121 ± 5	188 ± 6		*<75* 18.8	*76–84* 8.8	*85–89* 12	*>90* 59				
Scarfone et al. (2015) [37]	Amateur 10/male	166 ± 16	117 ± 17	188 ± 11	*<65* 8 ± 8	*66–75* 11 ± 7	*76–85* 27 ± 4	*86–95* 35 ± 12		*>95* 19 ± 7			6.7 ± 3.8
Bozdogan (2017) [36]	National 12/male	158 ± 11		181 ± 9									7.0 ± 2.6
Costa et al. (2022) [14]	International 11/male											*HI/RPE* 8 ± 2/7 ± 2	
***Beach handball***													
Pueo et al. (2017) [19]	International 12/male 12/female	137 ± 12 138 ± 18		174 ± 15 178 ± 15	*<60* 19 ± 17 27 ± 22	*61–70* 26 ± 12 16 ± 11	*71–80* 26 ± 12 18 ± 13	*81–90* 20 ± 14 29 ± 20	*91–95* 7 ± 10 8 ± 12	*>95* 2 ± 4 2 ± 6			
Gutiérrez-Vargas et al. (2019) [29]	National 8/male 8/female	156 ± 18 158 ± 20											
Zapardiel and Asín-Izquierdo (2020) [34]	International 25/male 32/female	150 ± 10 145 ± 18	129 ± 12 120 ± 23	164 ± 11 164 ± 16	*<60* ND ND	*60–70* ND ND	*70–80* ND ND	*80–90* 36 ± 27 36 ± 26	*>90* 1 ± 2 4 ± 7	*>95* ND ND	*TRIMPS* 65.6 ± 18.8 57.8 ± 20.4		
Iannaccone et al. (2021) [30]	International 13/male										*sHRZ* 77.4 ± 26.5	*RPE* 6 ± 1	
Sánchez-Sáez et al. (2021) [32]	International 9/female	170 ± 15		192 ± 13	*<60* 3 ± 7	*60–70* 9 ± 14	*70–80* 18 ± 15	*80–90* 33 ± 18	*90–95* 19 ± 15	*>95* 18 ± 24			
Gómez-Carmona et al. (2023) [15]	National 54/male 38/female	162 ± 23 150 ± 25			*<60* ND ND	*60–70* ND ND	*70–80* ND ND	*80–90* ND ND	*90–95* ND ND	*>95* ND ND			
Cobos et al. (2023) [27]	International 14/female				*<60* 26	*60–70* 10	*70–80* 17	*80–90* 29	*90–95* 12	*>95* 6			
Zapardiel et al. (2023) [33]	International 25/male 32/female	148 ± 14 147 ± 22	123 ± 21 122 ± 32	166 ± 14 168 ± 18	*<60* ND ND	*60–70* ND ND	*70–80* ND ND	*80–90* 35 ± 20 39 ± 17	*>90* 1 ± 2 2 ± 4	*>95* ND ND	*TRIMPS* 69.4 ± 19.9 58.9 ± 24.0		

**Note.** SD: standard deviation; bpm: beats per minute; a.u.: arbitrary units; HR: heart rate; HR_avg_: average heart rate; HR_min_: minimum heart rate; HR_max_: maximum heart rate; Z: Zone; TRIMPS: training impulses; sHRZ: summated heart rate zones; RPE: rate of perceived exertion (1–10 scale); HI: Hooper index; ND: no data.

## Data Availability

Not applicable.

## References

[1] Bělka J., Hůlka K., Šafář M., Weisser R., Chadimova J. (2015). Beach Handball and Beach Volleyball as Means Leading to Increasing Physical Activity of Recreational Sportspeople—Pilot Study. J. Sports Sci..

[2] Read B., Edwards P. (1992). Blue Section. Introducing Formal Games. Teaching Children to Play Games.

[3] Read B., Edwards P. (1992). Blue Section. Invasion Games. Teaching Children to Play Games.

[4] Hausler J., Halaki M., Orr R. (2016). Application of Global Positioning System and Microsensor Technology in Competitive Rugby League Match-Play: A Systematic Review and Meta-Analysis. Sports Med..

[5] Miguel M., Oliveira R., Loureiro N., García-Rubio J., Ibáñez S.J. (2021). Load Measures in Training/Match Monitoring in Soccer: A Systematic Review. Int. J. Environ. Res. Public Health.

[6] Petway A.J., Freitas T.T., Calleja-González J., Leal D.M., Alcaraz P.E. (2020). Training Load and Match-Play Demands in Basketball Based on Competition Level: A Systematic Review. PLoS ONE.

[7] Bourdon P.C., Cardinale M., Murray A., Gastin P., Kellmann M., Varley M.C., Gabbett T.J., Coutts A.J., Burgess D.J., Gregson W. (2017). Monitoring Athlete Training Loads: Consensus Statement. Int. J. Sports Physiol. Perform..

[8] Achenbach L., Krutsch W., Mayr H.O., Musahl V., Della Villa F., Tscholl P.M., Jones H. (2020). Beach Sports. Injury and Health Risk Management in Sports: A Guide to Decision Making.

[9] Impellizzeri F.M., Marcora S.M., Coutts A.J. (2019). Internal and External Training Load: 15 Years on. Int. J. Sports Physiol. Perform..

[10] Buchheit M., Lacome M., Cholley Y., Simpson B.M. (2018). Neuromuscular Responses to Conditioned Soccer Sessions Assessed via GPS-Embedded Accelerometers: Insights into Tactical Periodization. Int. J. Sports Physiol. Perform..

[11] Gómez-Carmona C.D., Bastida-Castillo A., Ibáñez S.J., Pino-Ortega J. (2020). Accelerometry as a Method for External Workload Monitoring in Invasion Team Sports. A Systematic Review. PLoS ONE.

[12] Foster C., Florhaug J.A., Franklin J., Gottschall L., Hrovatin L.A., Parker S., Doleshal P., Dodge C. (2001). A New Approach to Monitoring Exercise Training. J. Strength Cond. Res..

[13] Halson S.L. (2014). Monitoring Training Load to Understand Fatigue in Athletes. Sports Med..

[14] Costa J.A., Figueiredo P., Prata A., Reis T., Reis J.F., Nascimento L., Brito J. (2022). Associations between Training Load and Well-Being in Elite Beach Soccer Players: A Case Report. Int. J. Environ. Res. Public Health.

[15] Gómez-Carmona C.D., Rojas-Valverde D., Rico-González M., De Oliveira V., Lemos L., Martins C., Nakamura F., Pino-Ortega J. (2023). Crucial Workload Variables in Female-Male Elite Brazilian Beach Handball: An Exploratory Factor Analysis. Biol. Sport.

[16] Scott M.T.U., Scott T.J., Kelly V.G. (2016). The Validity and Reliability of Global Positioning Systems in Team Sport: A Brief Review. J. Strength Cond. Res..

[17] Bastida Castillo A., Gómez Carmona C.D., De la Cruz Sánchez E., Pino Ortega J. (2018). Accuracy, Intra- and Inter-Unit Reliability, and Comparison between GPS and UWB-Based Position-Tracking Systems Used for Time–Motion Analyses in Soccer. Eur. J. Sport Sci..

[18] Pino Ortega J., Rico González M. (2021). Standarization of Electronic Performance and Tracking Systems. The Use of Applied Technology in Team Sport.

[19] Pueo B., Jimenez-Olmedo J.M., Penichet-Tomas A., Becerra M.O., Agullo J.J.E. (2017). Analysis of Time-Motion and Heart Rate in Elite Male and Female Beach Handball. J. Sport Sci. Med..

[20] Bishop D. (2003). A Comparison between Land and Sand-Based Tests for Beach Volleyball Assessment. J. Sports Med. Phys. Fit..

[21] Castellano J., Casamichana D. (2010). Heart Rate and Motion Analysis by GPS in Beach Soccer. J. Sports Sci. Med..

[22] Ato M., López-García J.J., Benavente A. (2013). Un Sistema de Clasificación de Los Diseños de Investigación en Psicología. An. Psicol..

[23] Page M.J., McKenzie J.E., Bossuyt P.M., Boutron I., Hoffmann T.C., Mulrow C.D., Shamseer L., Tetzlaff J.M., Akl E.A., Brennan S.E. (2021). The PRISMA 2020 Statement: An Updated Guideline for Reporting Systematic Reviews. Int. J. Surg..

[24] Rico-González M., Pino-Ortega J., Clemente F., Los Arcos A. (2022). Guidelines for Performing Systematic Reviews in Sports Science. Biol. Sport.

[25] Moher D., Shamseer L., Clarke M., Ghersi D., Liberati A., Petticrew M., Shekelle P., Stewart L.A. (2015). Preferred Reporting Items for Systematic Review and Meta-Analysis Protocols (PRISMA-P) 2015 Statement. Syst. Rev..

[26] Slim K., Nini E., Forestier D., Kwiatkowski F., Panis Y., Chipponi J. (2003). Methodological Index for Non-randomized Studies (*MINORS*): Development and Validation of a New Instrument. ANZ J. Surg..

[27] Cobos D.L., Ortega-Becerra M., Daza G., Sánchez-Sáez J.A. (2023). Internal and External Load in International Women’s Beach Handball: Official and Unofficial Competition. Apunts Educ. Física Esports.

[28] Gómez-Carmona C.D., García-Santos D., Mancha-Triguero D., Antúnez A., Ibáñez S.J., Gómez-Carmona C.D., García-Santos D., Mancha-Triguero D., Antúnez A., Ibáñez S.J. (2020). Analysis of Sex-Related Differences in External Load Demands on Beach Handball. Braz. J. Kinanthropometry Hum. Perform..

[29] Gutiérrez-Vargas R., Gutiérrez-Vargas J.C., Ugalde-Ramírez J.A., Rojas-Valverde D. (2019). Kinematics and Thermal Sex-Related Responses during an Official Beach Handball Game in Costa Rica: A Pilot Study. Arch. Med. Deporte.

[30] Iannaccone A., Fusco A., Skarbalius A., Kniubaite A., Cortis C., Conte D. (2021). Relationship between External and Internal Load Measures in Youth Beach Handball. Int. J. Sports Physiol. Perform..

[31] Müller C., Willberg C., Reichert L., Zentgraf K. (2022). External Load Analysis in Beach Handball Using a Local Positioning System and Inertial Measurement Units. Sensors.

[32] Sánchez-Sáez J.A., Sánchez-Sánchez J., Martínez-Rodríguez A., Felipe J.L., García-Unanue J., Lara-Cobos D. (2021). Global Positioning System Analysis of Physical Demands in Elite Women’s Beach Handball Players in an Official Spanish Championship. Sensors.

[33] Zapardiel J.C., Paramio E.M., Ferragut C., Vila H., Asin-Izquierdo I. (2023). Comparison of Internal and External Load Metrics between Won and Lost Game Segments in Elite Beach Handball. Hum. Mov..

[34] Zapardiel J.C., Asín-Izquierdo I. (2020). Conditional Analysis of Elite Beach Handball According to Specific Playing Position through Assessment with GPS. Int. J. Perform. Anal. Sport.

[35] Mancha-Triguero D., González-Espinosa S., Córdoba L.G., García-Rubio J., Feu S. (2020). Differences in the Physical Demands between Handball and Beach Handball Players. Rev. Bras. Cineantropometria Desempenho Hum..

[36] Bozdogan T.K. (2017). Evaluate the Physical and Physiological Characteristics of Turkish National Beach Soccer Players. Acta Sci. Intellectus.

[37] Scarfone R., Tessitore A., Minganti C., Capranica L., Ammendolia A. (2015). Match Analysis Heart-Rate and CMJ of Beach Soccer Players during Amateur Competition. Int. J. Perform. Anal. Sport.

[38] Fédération Internationale de Football Association Beach Soccer Laws of the Game 2023-2024 2023.

[39] International Handball Federation Rules of the Game for Beach Handball 2021.

[40] Achenbach L., Loose O., Laver L., Zeman F., Nerlich M., Angele P., Krutsch W. (2018). Beach Handball Is Safer than Indoor Team Handball: Injury Rates during the 2017 European Beach Handball Championships. Knee Surg. Sports Traumatol. Arthrosc..

[41] Reardon C., Tobin D.P., Delahunt E. (2015). Application of Individualized Speed Thresholds to Interpret Position Specific Running Demands in Elite Professional Rugby Union: A GPS Study. PLoS ONE.

[42] Abt G., Lovell R. (2009). The Use of Individualized Speed and Intensity Thresholds for Determining the Distance Run at High-Intensity in Professional Soccer. J. Sports Sci..

[43] Clemente F., Ramirez-Campillo R., Beato M., Moran J., Kawczynski A., Makar P., Sarmento H., Afonso J. (2023). Arbitrary Absolute vs. Individualized Running Speed Thresholds in Team Sports: A Scoping Review with Evidence Gap Map. Biol. Sport.

[44] Polglaze T., Hogan C., Dawson B., Buttfield A., Osgnach C., Lester L., Peeling P. (2018). Classification of Intensity in Team Sport Activity. Med. Sci. Sports Exerc..

[45] Sweeting A.J., Cormack S.J., Morgan S., Aughey R.J. (2017). When Is a Sprint a Sprint? A Review of the Analysis of Team-Sport Athlete Activity Profile. Front. Physiol..

[46] Ibáñez S.J., Gómez-Carmona C.D., Mancha-Triguero D. (2022). Individualization of Intensity Thresholds on External Workload Demands in Women’s Basketball by K-Means Clustering: Differences Based on the Competitive Level. Sensors.

[47] Spyrou K., Freitas T.T., Marín-Cascales E., Alcaraz P.E. (2020). Physical and Physiological Match-Play Demands and Player Characteristics in Futsal: A Systematic Review. Front. Psychol..

[48] García-Sánchez C., Navarro R.M., Karcher C., de la Rubia A. (2023). Physical Demands during Official Competitions in Elite Handball: A Systematic Review. Int. J. Environ. Res. Public Health.

[49] Eils E., Wirtz S., Brodatzki Y., Zentgraf K., Büsch D., Szwajca S. (2022). Optimizing the Transition from the Indoor to the Beach Season Improves Motor Performance in Elite Beach Handball Players. Ger. J. Exerc. Sport Res..

[50] Balasas D., Vamvakoudis E., Christoulas K., Stefanidis P., Prantsidis D., Evangelia P. (2013). The Effect of Beach Volleyball Training on Running Economy and VO2max of Indoor Volleyball Players. J. Phys. Educ. Sport.

[51] Malone J.J., Lovell R., Varley M.C., Coutts A.J. (2017). Unpacking the Black Box: Applications and Considerations for Using GPS Devices in Sport. Int. J. Sports Physiol. Perform..

[52] Gómez Carmona C.D., Pino Ortega J., Rico González M., Bastida Castillo A. (2021). Global Navigation Satellite Systems. The Use of Applied Technology in Team Sport.

[53] Edwards S., White S., Humphreys S., Robergs R., O’Dwyer N. (2019). Caution Using Data from Triaxial Accelerometers Housed in Player Tracking Units during Running. J. Sports Sci..

[54] Camomilla V., Bergamini E., Fantozzi S., Vannozzi G. (2018). Trends Supporting the In-Field Use of Wearable Inertial Sensors for Sport Performance Evaluation: A Systematic Review. Sensors.

[55] Aughey R.J., Falloon C. (2010). Real-Time versus Post-Game GPS Data in Team Sports. J. Sci. Med. Sport.

[56] Chambers R., Gabbett T.J., Cole M.H., Beard A. (2015). The Use of Wearable Microsensors to Quantify Sport-Specific Movements. Sports Med..

